# The Association Between Personality Traits and Health-Related Quality of Life and the Mediating Role of Smoking: Nationwide Cross-Sectional Study

**DOI:** 10.2196/51416

**Published:** 2024-07-05

**Authors:** Jiangyun Chen, Jiahuan Wan, Yibo Wu, Li Gan, Haomiao Li, Yan Zhou, Siyuan Liu, Lan Luo, Haozheng Zhou, Xuanhao Yin, Jinghui Chang

**Affiliations:** 1 Center for WHO Studies and Department of Health Management, School of Health Management, Southern Medical University, Guangzhou, China; 2 School of Political Science and Public Administration, Wuhan University, Wuhan, China; 3 Operation Management Department, Zhuhai People's Hospital (Zhuhai Hospital Affiliated with Jinan University), Zhuhai, China; 4 School of Public Health, Southern Medical University, Guangzhou, China; 5 Guangzhou Huangpu District Hongshan Street Community Health Service Center, Guangzhou, China

**Keywords:** Big Five personality, HRQOL, smoking, mediation, tobacco control, China, neuroticism, extraversion, agreeableness, health-related quality of life

## Abstract

**Background:**

There are positive and negative correlations in different directions between smoking, personality traits, and health-related quality of life (HRQOL), where smoking may mask the pathway between personality traits and HRQOL. Understanding the masking pathway of smoking between personality traits and HRQOL can elucidate the mechanisms of smoking’s psychosocial effects and provide new ideas for developing tobacco control strategies.

**Objective:**

The purpose of this study was to investigate the correlation between Big Five personality traits and HRQOL and whether smoking mediates the relationship between them.

**Methods:**

This was a cross-sectional study using data from 21,916 respondents from the 2022 Psychology and Behavior Investigation of Chinese Residents survey. Linear regression models were used to analyze the correlations between smoking, Big Five personality traits, and HRQOL while controlling for potential confounders. The mediating role of smoking on the association between Big Five Personality traits and HRQOL was analyzed using the Sobel-Goodman mediation test.

**Results:**

Extraversion (β=.001; *P*=.04), agreeableness (β=.003; *P*<.001), and neuroticism (β=.003; *P*<.001) were positively correlated with HRQOL, whereas openness was negatively correlated with HRQOL (β=–.001; *P*=.003). Smoking was associated with a decrease in HRQOL and mediated the positive effect of HRQOL on extraversion (*z*=−2.482; *P*=.004), agreeableness (*z*=−2.264; *P*=.02), and neuroticism (*z*=−3.230; *P*=.001). Subgroup analyses further showed that smoking mediated the effect of neuroticism on HRQOL in the population with chronic illnesses (*z*=−2.724; *P*=.006), and in the population without chronic illnesses, smoking contributed to the effect of HRQOL on extraversion (*z*=−2.299; *P*=.02), agreeableness (*z*=−2.382; *P*=.02), and neuroticism (*z*=−2.213; *P*=.03).

**Conclusions:**

This study provided evidence that there is a correlation between personality traits and HRQOL. It also found that smoking plays a role in mediating the connection between personality traits and HRQOL. The development of future tobacco control strategies should consider the unique traits of each individual’s personality, highlighting the significance of extraversion, agreeableness, and neuroticism.

## Introduction

A widely recognized theoretical framework in the field of personality psychology is the Big Five personality model, which classifies personality traits into 5 dimensions: openness, conscientiousness, extraversion, agreeableness, and neuroticism [1]. The stability and validity of the Big Five personality theory have been consistently confirmed by previous research, which has conducted long-term follow-up studies, cross-cultural studies, and comparisons across various age groups. Studies have revealed that the Big Five personality traits are significantly correlated with diverse behaviors and adaptations [2], along with their interplay with genetic and environmental factors [3,4]. Various characteristics of individuals have been discovered to have a strong association with academic success [5], professional pathways [6], interpersonal connections [7], physical well-being, and psychological well-being [8,9]. The extensive applications of the Big Five personality theory span across multiple domains, encompassing talent management [10], mental health research [11], style of behavior [11,12], clinical practice [13], personal growth, and education [14,15]. By evaluating the Big Five personality traits, scientists have gained insights into individuals’ personality characteristics, offering direction and support. Prior research has additionally indicated that although a person’s character attributes remain relatively consistent, the manifestation of these attributes is not completely rigid. Moreover, an individual’s encounters, surroundings, and cultural upbringing can also influence their character traits and their overall quality of life in terms of health (health-related quality of life [HRQOL]) [16,17].

The Big Five personality theory is extensively used in the domains of health behavior and psychology to examine how personality traits influence behavioral traits and health outcomes in populations [18,19]. Research has confirmed a robust correlation between aspects of the Big Five personality traits and HRQOL. People who have a strong inclination toward openness are generally more welcoming toward novel encounters, have a wider array of hobbies, and exhibit a sense of curiosity. These traits are linked to increased levels of contentment and overall quality of life [20]. Individuals who possess a strong sense of duty and a proactive attitude toward accomplishing tasks tend to have high conscientiousness. This trait is linked to improved mental well-being, reduced negative emotions, and enhanced HRQOL [21]. Individuals who have a high level of extraversion generally have enhanced social abilities, display optimistic emotional expression, and are more inclined to form and sustain positive interpersonal connections. As a result, they experience increased life satisfaction and happiness [22]. People who possess a strongly agreeable nature tend to pay closer attention to the happiness and welfare of those around them. They demonstrate kindness, empathy, and assistance in social engagements, which are linked to improved HRQOL, increased social support, and reduced feelings of isolation [23]. Individuals exhibiting elevated levels of neuroticism are prone to experience anxiety, tension, and negative emotions, which have been linked to diminished levels of life contentment and overall welfare [24]. However, some studies have proposed the concept of “healthy neuroticism,” which refers to individuals with neurotic traits that do not lead to physical and mental health problems [25,26]. The healthy neuroticism theory suggests that neurotic individuals may pursue perfection, possess higher alertness and introspection, and have higher demands on themselves, which may positively influence health behaviors and thus positively impact HRQOL through high alertness to unhealthy behaviors [27].

Personality is also associated with smoking. For example, people with a high level of extraversion are usually more prone to seeking excitement and engaging in daring pursuits, which increases their susceptibility to the temptation of smoking [28]. On the other hand, people with a strong sense of conscientiousness are inclined to be more accountable and have a greater tendency to follow healthy habits, such as refraining from smoking [29]. People with a high level of agreeableness typically exhibit a compliant and cooperative demeanor. As such, those with higher agreeableness exhibit more positive social and prosocial behaviors [30], but they are also more susceptible to being socially influenced to smoke. People with a high level of openness tend to be more inclined to engage in novel activities and are more susceptible to experimenting with smoking [31]. In contrast, people with a high level of neuroticism are prone to feeling anxious and stressed and experiencing negative emotions, which increases their likelihood of initiating smoking and makes it harder for them to quit [32].

Older adults and individuals with underlying diseases are particularly affected by smoking, as it leads to a decline in HRQOL due to health hazards, mental strain, financial strain, and limitations on social activities [33,34]. Multiple research studies have firmly established a robust correlation between tobacco use and the emergence of diverse ailments, such as increasing the burden of cervical cancer and mortality [35], underscoring its substantial capacity to jeopardize human well-being. According to the *World Health Organization Report on the Global Tobacco Epidemic 2021*, tobacco remains a significant contributor to untimely fatalities on a global scale [36]. China, being the biggest manufacturer and user of tobacco on a global scale, bears the sole responsibility for around 1 million tobacco-related deaths [37]. The use of tobacco is a significant contributing factor linked to the greatest load of long-term illness, and a decrease in tobacco consumption can result in decreased occurrences of heart disease, stroke, and additional chronic ailments.

Past studies have confirmed that characteristics of an individual’s personality affect both their HRQOL and smoking habits, where smoking is found to have an adverse effect on HRQOL. Nevertheless, the correlation among these 3 variables remains incompletely comprehended, particularly concerning individuals with long-term illnesses. Examining the mechanisms of interaction between Big Five personality traits, smoking, and HRQOL and conducting tobacco control efforts at the level of individual personality traits can provide new perspectives for improving HRQOL. Based on these reasons, this study formulated the following hypotheses:

- Hypothesis 1: The Big Five personality traits influence HRQOL.
- Hypothesis 2: Smoking is associated with Big Five personality traits and HRQOL, where smoking plays a mediating role between them.

## Methods

### Participants

The information used in this investigation was acquired from the 2022 Psychology and Behavior Investigation of Chinese Residents survey. From June to August 2022, a comprehensive survey was carried out in 148 cities; 202 districts and counties; 390 townships, towns, and streets; and 780 communities and villages spanning 23 provinces, 5 autonomous regions, and 4 municipalities under the central government. To ensure the overall representativeness of the study population, the survey used a multistage sampling technique, incorporating stratified sampling at various levels including provincial; city; district and county; township, town, and street; and community and village. Quota sampling was used at the community and village level as well as at the individual level, using quotas that were determined based on sex and age attributes from the data of the Seventh National Population Census. In every city, there was recruitment of at least 1 enumerator or survey team, where each enumerator had the duty of gathering 30‐90 questionnaires and each survey team had the duty of gathering 100‐200 questionnaires. The questionnaires were distributed through the web-based Questionnaire Star platform, and if face-to-face surveys were possible in the area, the investigator filled out the questionnaires on site on a one-to-one basis. In the event that face-to-face surveys were impractical due to the constraints of the new coronavirus outbreak, electronic surveys were individually provided to the participants. Participants provided their responses by clicking on the questionnaire link, and they were required to give their informed consent. The study included individuals who were at least 12 years of age, held citizenship in the People’s Republic of China, and were permanent residents of China with an annual out-of-home time of no more than 1 month. Participants who did not fulfill the criteria for this research were disqualified. A total of 23,414 questionnaires were collected for the study, ensuring high quality and national representativeness of the data. After eliminating duplicates and excluding missing data and logically inconsistent outlier data, 21,916 respondents were finally included in this study, with a valid response rate of 93.6% (21,916/23,414). The survey protocol has been published [38].

### Ethical Considerations

This study complied with the ethical review rules of the Health Culture Research Center of Shaanxi (JKWH-2022-02). Informed consent was obtained for the investigation. Respondents completed an anonymous, web-based survey in approximately 30 minutes.

### Variables

#### Dependent Variable

The health status of the population was assessed using HRQOL in this study. The measurement of HRQOL was conducted using the EQ-5D-5L traditional scale, which has been proven to be better than its previous version, the EQ-5D-3L, in terms of practicality, upper limit impact, distinguishing ability, and agreement with other measures [39,40]. The EQ-5D-5L scale consists of 5 aspects: mobility, self-care, daily activity performance, pain or discomfort, and anxiety or depression, as specified in Multimedia Appendix 1. There are 5 levels for each dimension, ranging from 1 (no problems) to 5 (extreme problems). The levels of these questions can describe 243 different health states, forming different outcomes for combinations ranging from 11,111 (perfectly good) to 55,555 (perfectly poor). These health states are assigned an index value, known as the health state index (HIS), which reflects the weighting of society’s preference for the health state. The HIS score varied from below 0 (where 0 represents the health state value of death; negative values indicate a health state worse than death) to 1 (representing perfect health), with higher scores indicating better health utility [40]. Based on their health preferences, the HIS value estimates for the Chinese population vary between −0.391 and 1, representing the worst and best outcomes. In this study, the final HIS was obtained according to the utility value conversion formula *(X – min) / (max – min)* of [0,1] [41].

#### Independent Variable

The assessment of personality involved the use of the Big Five Inventory (BFI)–10, a condensed variant of the comprehensive BFI-44. In this study, the reliability and validity of the BFI-10 were assessed to confirm its suitability for situations where there are time constraints or it is not feasible to conduct a personality assessment (eg, telephone surveys, etc). The scale consists of 10 items that assess 5 personality dimensions: extraversion, agreeableness, conscientiousness, neuroticism, and openness. A 5-point Likert scale is used to score each item, with higher scores indicating a stronger trait. Extraversion is assessed by questions 1 and 6, agreeableness is assessed by questions 2 and 7, conscientiousness is assessed by questions 3 and 8, neuroticism is assessed by questions 4 and 9, and openness is assessed by questions 5 and 10. It should be mentioned that questions 1, 3, 5, 7, and 9 are scored in the opposite direction [42].

#### Mediation Variables

Smoking behavior was assessed by asking respondents about their current smoking habits. Specifically, they were asked, “Do you currently smoke?” Responses were categorized into 5 levels: 0=“No”; 1=“Yes, regular cigarettes”; 2=“Yes, e-cigarettes”; 3=“Yes, both”; and 4=“Ever (quit).” For this study, smoking was divided into 2 categories depending on whether participants were presently smoking or not: 0=never smoked or used to smoke but have stopped, and 1=currently smoking.

### Covariates

The variables examined in this research consist of the socioeconomic background of the participants (including sex, age group, area of residence, ethnicity, political status, religion, household income, educational level, occupation, and social status); family attributes (marital status and family type); lifestyle elements (smoking habits and alcohol intake); and mental health status related to perception of stress, perception of social support, self-confidence, and health knowledge. Detailed information on the definitions and categorization of these variables can be found in Multimedia Appendix 2. The choice of covariates was determined by their correlation with the independent variables, as well as their impact on the association between the independent variables and the dependent variable. Age group and sex were adjusted as fixed covariates. If the other covariates changed the dependent variable by more than 10% with the independent variable or were significantly associated with the dependent variable, they were included as potential confounding factors in the final model. The Empower software (X&Y Solutions) was used to test the selected covariates, which were chosen based on established associations or plausible biological relationships. These covariates include ethnicity, political status, religion, area of residence (a special Chinese identifier that impacts various aspects of life in China, such as purchasing a house or a car, public health insurance reimbursement rate, and welfare benefits), household income, education level, occupation, social status, marital status, family type, alcohol consumption, stress perception ability, social support appreciation ability, self-efficacy, and health literacy. Multimedia Appendices 3 and 4 contain detailed findings.

### Statistical Analysis

The basic study population description included the presentation of chronic diseases characteristics (yes, no, and total) as mean (SD) for continuous variables and as frequency (%) for categorical variables. To examine variations in the attributes of chronic diseases, a 2-tailed Student *t* test was used for continuous factors, whereas the *χ*^*2*^ test was used for categorical factors. The correlation between smoking, Big Five personality traits, and HRQOL was measured using linear regression models. This was done before and after adjusting for covariates, and the findings were presented as β coefficients along with 95% CIs. The Sobel-Goodman mediation test was used to examine the impact of smoking on Big Five personality traits and HRQOL while taking into account all covariates [43]. Statistical significance was determined using 2-sided *P* values, with α<.05 as the threshold. The analysis of data was conducted using Stata (version 17; StataCorp).

## Results

### General Characteristics

The sample analyzed in this study consisted of 21,916 cases. There was an equal distribution of sexes, with 10,958 (50%) participants identifying as male and 10,958 (50%) identifying as female. A total of 71.4% (n=15,647) of the participants fell within the age group of 18‐59 years, and 56.75% (n=12,437) of them were married. The vast majority of respondents were of Han nationality (n=19,970, 91.12%), had no religion (n=21,058, 96.09%), and had “the masses” as their political status (n=13,912, 63.48%). Over half (n=11,811, 53.89%) of the participants lived in urban regions, with a greater proportion belonging to the high-income bracket (n=8032, 36.65%). The majority (n=15,214, 69.42%) of the respondents reported never drinking alcohol, and the largest proportion (n=9773, 44.59%) had tertiary education. In all, 34.68% (n=7601) were employed, with over half (n=11,574, 52.81%) of the family type being a core family. The prevalence of smoking was 14.87% (n=3258). The average social status of the respondents was close to the upper-middle class (mean 4.35, SD 1.30; out of a total score of 6). Among individuals with chronic illnesses, there was a notable decline in HRQOL (mean 0.92, SD 0.13), which was significantly lower than that of the overall sample (mean 0.96, SD 0.10; *P*<.001). Individuals with chronic illnesses exhibited a diminished level of extraversion (mean 6.14, SD 1.61; *P*<.001) compared to that of the overall sample (mean 6.23, SD 1.62). The average rating for agreeableness was 7.00 (SD 1.48). The average score for conscientiousness was 6.76 (SD 1.65), and individuals with chronic diseases exhibited a higher level of conscientiousness (mean 6.98, SD 1.65; *P*<.001). The average score for neuroticism was 6.27 (SD 1.56). Individuals with chronic illnesses exhibited a diminished level of openness (mean 6.20, SD 1.61), which was significantly lower than that of the overall sample (mean 6.46, SD 1.55; *P*<.001). The average rating for perceived stress capacity was 6.55 (SD 2.54), indicating an increase among individuals with chronic illnesses (mean 6.63, SD 2.57; *P*=.02). Moreover, the population with chronic illnesses experienced a decrease in their corresponding competencies, specifically in comprehending social support (mean 15.03, SD 3.78), self-efficacy (mean 7.79, SD 2.42), and health literacy (mean 27.55, SD 5.30), suggesting a decline in these abilities (*P*<.001). Table 1 contains comprehensive details.

**Table 1.** Characteristics of respondents^a^.

Characteristics		Overall (N=21,916)	With chronic disease (n=5460)	Without chronic disease (n=21,796)	*P* value
**Age group (years), n (%)**					<.001
	12‐17	2072 (9.45)	136 (2.49)	1936 (11.76)	
	18‐59	15,647 (71.4)	2943 (53.9)	12,704 (77.2)	
	≥60	4197 (19.15)	2381 (43.61)	1816 (11.04)	
**Sex, n (%)**					<.001
	Male	10,958 (50)	2854 (52.27)	8104 (49.25)	
	Female	10,958 (50)	2606 (47.73)	8352 (50.75)	
**Marital status, n (%)**					<.001
	Never married	8497 (38.77)	851 (15.59)	7646 (46.46)	
	Married	12,437 (56.75)	4059 (74.34)	8378 (50.91)	
	Divorce	406 (1.85)	160 (2.93)	246 (1.49)	
	Widowed	576 (2.63)	390 (7.14)	186 (1.13)	
**Ethnicity, n (%)**					.86
	Han nationality	19,970 (91.12)	4972 (91.06)	14,998 (91.14)	
	Ethnic minority	1946 (8.88)	488 (8.94)	1458 (8.86)	
**Religion, n (%)**					<.001
	None	21,058 (96.09)	5091 (93.24)	15,967 (97.03)	
	Yes	858 (3.91)	369 (6.76)	489 (2.97)	
**Political status, n (%)**					<.001
	Party member or probationary party member	3179 (14.51)	1059 (19.4)	2120 (12.88)	
	Member of the Communist Youth League	4671 (21.31)	457 (8.37)	4214 (25.61)	
	Other parties	154 (0.7)	59 (1.08)	95 (0.58)	
	The masses	13,912 (63.48)	3885 (71.15)	10,027 (60.93)	
**Area of residence, n (%)**					.007
	Urban	11,811 (53.89)	2856 (52.31)	8955 (54.42)	
	Rural	10,105 (46.11)	2604 (47.69)	7501 (45.58)	
**Family income, n (%)**					<.001
	Low	7229 (32.99)	2050 (37.55)	5179 (31.47)	
	Moderate	6655 (30.37)	1628 (29.82)	5027 (30.55)	
	High	8032 (36.65)	1782 (32.64)	6250 (37.98)	
**Alcohol intake, n (%)**					<.001
	Never	15,214 (69.42)	3277 (60.02)	11,937 (72.54)	
	All the time	3266 (14.9)	1019 (18.66)	2247 (13.65)	
	Used to drink, but does not drink now	2148 (9.80)	947 (17.34)	1201 (7.3)	
	Did not drink in the past, but drinks now	1288 (5.88)	217 (3.97)	1071 (6.51)	
**Education level, n (%)**					<.001
	Primary school and below	3412 (15.57)	1528 (27.99)	1884 (11.45)	
	Middle school and junior college	8731 (39.84)	1977 (36.21)	6754 (41.04)	
	College degree or above	9773 (44.59)	1955 (35.81)	7818 (47.51)	
**Occupation, n (%)**					<.001
	Employed	7601 (34.68)	1633 (29.91)	5968 (36.27)	
	Student	6580 (30.02)	557 (10.2)	6023 (36.6)	
	Retirement	2756 (12.58)	1539 (28.19)	1217 (7.4)	
	No regular occupation	2609 (11.9)	707 (12.95)	1902 (11.56)	
	Unemployed	2370 (10.81)	1024 (18.75)	1346 (8.18)	
**Family type^b^, n (%)**					<.001
	Backbone family	3717 (16.96)	989 (18.11)	2728 (16.58)	
	Core family	11,574 (52.81)	1922 (35.2)	9652 (58.65)	
	Conjugal family	3836 (17.5)	1639 (30.02)	2197 (13.35)	
	Other	2789 (12.73)	910 (16.67)	1879 (11.42)	
**Smoking habit, n (%)**					<.001
	No	18,658 (85.13)	4306 (78.86)	14,352 (87.21)	
	Yes	3258 (14.87)	1154 (21.14)	2104 (12.79)	
Health-related quality of life, mean (SD)		0.96 (0.10)	0.92 (0.13)	0.97 (0.09)	<.001
Extraversion, mean (SD)		6.23 (1.62)	6.14 (1.61)	6.27 (1.62)	<.001
Agreeableness, mean (SD)		7.00 (1.48)	6.98 (1.52)	7.00 (1.47)	.27
Conscientiousness, mean (SD)		6.76 (1.65)	6.98 (1.65)	6.69 (1.64)	<.001
Neuroticism, mean (SD)		6.27 (1.56)	6.25 (1.60)	6.28 (1.54)	.19
Openness, mean (SD)		6.46 (1.55)	6.20 (1.61)	6.55 (1.52)	<.001
Health literacy, mean (SD)		27.55 (5.30)	26.29 (5.45)	27.96 (5.19)	<.001
Self-efficacy, mean (SD)		7.79 (2.42)	7.55 (2.47)	7.87 (2.40)	<.001
Perceived stress, mean (SD)		6.55 (2.54)	6.63 (2.57)	6.52 (2.53)	.02
Perceived social support, mean (SD)		15.03 (3.78)	14.63 (3.74)	15.16 (3.79)	<.001
Social status, mean (SD)		4.35 (1.30)	4.30 (1.28)	4.36 (1.31)	.003

a Mean (SD) was used to describe continuous variables, and frequency (%) was used to describe categorical variables.

b “Backbone family” refers to a family consisting of 2 spouses—a husband and a wife. “Core family” refers to a family consisting of parents and unmarried children. “Conjugal family” refer to a family consisting of parents and married children. “Other” consists of the following: families consisting of parents and more than 2 married children or siblings married without joint families; single-parent families; DINK; intergenerational families; single families; reconstituted families; cohabiting families; homosexual families, etc.

### Correlation Analysis

Before accounting for covariates, the linear regression model revealed a detrimental association between smoking and HRQOL (β=–.028; *P*<.001). Additionally, HRQOL exhibited significant correlations with extraversion (*P*<.001), agreeableness (*P*<.001), conscientiousness (*P*<.001), neuroticism (*P*<.001), and openness (*P*=.001). After making adjustments for various factors such as sex, age range, ethnicity, political status, religion, area of residence, household income, place of residence, education level, occupation, social status, marital status, family structure, alcohol consumption, ability to perceive stress, ability to appreciate social support, self-confidence, and health knowledge, the findings indicated that smoking still had a negative association with HRQOL (β=–.016; *P*<.001). Additionally, extraversion (β=.001; *P*=.04), agreeableness (β=.003; *P*<.001), neuroticism (β=.003; *P*<.001), and openness (β=–.001; *P*=.003) were all significantly linked to HRQOL. However, in the population with chronic illnesses, the findings of the model, after accounting for covariates, indicated that only tobacco use (β=–.021; *P*<.001), agreeableness (β=.003; *P*=.005), and neuroticism (β=.005; *P*<.001) exhibited a correlation with HRQOL. Table 2 provides the comprehensive details.

Before accounting for covariates, the initial linear regression analysis revealed that smoking had a negative correlation with agreeableness (β=−.107; *P*<.001), neuroticism exhibited a positive correlation with smoking (β=.185; *P*<.001), and openness displayed a negative correlation with smoking (β=−.247; *P*<.001). The findings after accounting for covariates indicated that extraversion (β=.077; *P*=.02), agreeableness (β=.059; *P*=.04), and neuroticism (β=.089; *P*=.004) exhibited a positive association with smoking. After accounting for covariates, the model demonstrated a noteworthy impact of neuroticism (β=.155; *P*=.004) within the group of individuals with chronic illnesses. Table 3 provides a comprehensive overview of the detailed information.

**Table 2.** Linear regression analysis for the associations of health-related quality of life (HRQOL) with smoking and Big Five personality traits.

Factor	Overall (N=21,916)				With chronic disease (n=5460)				Without chronic disease (n=21,796)			
	Unadjusted, β^a^ (95% CI)	*P* value	Adjusted^b^, β (95% CI)	*P* value	Unadjusted, β (95% CI)	*P* value	Adjusted^c^, β (95% CI)	*P* value	Unadjusted, β (95% CI)	*P* value	Adjusted^c^, β (95% CI)	*P* value
Smoking	−0.028 (−0.032 to −0.024)	<.001	−0.016 (−0.020 to −0.012)	<.001	−0.035 (−0.043 to −0.027)	<.001	−0.021 (−0.029 to −0.012)	<.001	−0.016 (−0.020 to −0.013)	<.001	−0.010 (−0.014 to −0.005)	<.001
Extraversion	0.004 (0.003 to 0.005)	<.001	0.001 (−0.000 to 0.002)	.04	0.006 (0.004 to 0.008)	<.001	0.001 (−0.001 to 0.003)	.22	0.003 (0.002 to 0.004)	<.001	0.001 (−0.000 to 0.001)	.19
Agreeableness	0.008 (0.007 to 0.009)	<.001	0.003 (0.002 to 0.003)	<.001	0.012 (0.010 to 0.014)	<.001	0.003 (0.001 to 0.005)	.005	0.007 (0.006 to 0.008)	<.001	0.002 (0.002 to 0.003)	<.001
Conscientiousness	0.006 (0.005 to 0.006)	<.001	0.001 (−0.000 to 0.001)	.19	0.010 (0.008 to 0.012)	<.001	0.001 (−0.001 to 0.003)	.50	0.005 (0.005 to 0.006)	<.001	0.001 (−0.000 to 0.002)	.13
Neuroticism	0.008 (0.007 to 0.009)	<.001	0.003 (0.003 to 0.004)	<.001	0.011 (0.009 to 0.013)	<.001	0.005 (0.003 to 0.007)	<.001	0.007 (0.006 to 0.007)	<.001	0.003 (0.002 to 0.004)	<.001
Openness	0.001 (0.001 to 0.002)	.001	−0.001 (−0.002 to −0.000)	.003	0.002 (0.000 to 0.005)	.02	0.000 (−0.002 to 0.002)	.78	−0.001 (−0.001 to 0.000)	.15	−0.002 (−0.003 to −0.001)	<.001

a β: beta coefficient.

b Adjusting for sex, age group, marital status, ethnicity, religion, political status, chronic disease, area of residence, family income, alcohol intake, education level, occupation, social status, family type, health literacy, self-efficacy, perceived stress, and perceived social support.

c Adjusting for sex, age group, marital status, ethnicity, religion, political status, area of residence, family income, alcohol intake, education level, occupation, social status, family type, health literacy, self-efficacy, perceived stress, and perceived social support.

**Table 3.** Linear regression analysis for the associations of smoking with Big Five personality traits.

Factor	Overall (N=21,916)				With chronic disease (n=5460)				Without chronic disease (n=21,796)			
	Unadjusted, β^a^ (95% CI)	*P* value	Adjusted^b^, β (95% CI)	*P* value	Unadjusted, β (95% CI)	*P* value	Adjusted^c^, β (95% CI)	*P* value	Unadjusted, β (95% CI)	*P* value	Adjusted^c^, β (95% CI)	*P* value
Extraversion	−0.026 (−0.086 to 0.034)	.40	0.077 (0.011 to 0.142)	.02	−0.088 (−0.193 to 0.016)	.10	0.106 (−0.006 to 0.219)	.06	0.028 (−0.046 to 0.102)	.46	0.071 (−0.011 to 0.152)	.09
Agreeableness	−0.107 (−0.162 to −0.052)	<.001	0.059 (−0.000 to 0.118)	.04	−0.201 (−0.300 to −0.103)	<.001	0.043 (−0.059 to 0.146)	.41	−0.058 (−0.125 to 0.010)	.09	0.074 (0.002 to 0.147)	.04
Conscientiousness	0.037 (−0.024 to 0.098)	.24	−0.015 (−0.076 to 0.046)	.63	−0.309 (−0.417 to −0.202)	<.001	−0.047 (−0.153 to 0.059)	.38	0.156 (0.081 to 0.231)	<.001	0.014 (−0.061 to 0.090)	.71
Neuroticism	0.185 (0.128 to 0.243)	<.001	0.089 (0.028 to 0.150)	.004	0.086 (−0.018 to 0.190)	.11	0.155 (0.049 to 0.260)	.004	0.243 (0.173 to 0.314)	<.001	0.063 (−0.012 to 0.137)	.10
Openness	−0.247 (−0.305 to −0.189)	<.001	0.037 (−0.025 to 0.098)	.24	−0.004 (−0.109 to 0.101)	.94	0.092 (−0.018 to 0.202)	.10	−0.306 (−0.376 to −0.237)	<.001	−0.007 (−0.082 to 0.068)	.86

a β: beta coefficient

b Adjusting for sex, age group, marital status, ethnicity, religion, political status, chronic disease, area of residence, family income, alcohol intake, education level, occupation, social status, family type, health literacy, self-efficacy, perceived stress, and perceived social support.

c Adjusting for sex, age group, marital status, ethnicity, religion, political status, area of residence, family income, alcohol intake, education level, occupation, social status, family type, health literacy, self-efficacy, perceived stress, and perceived social support.

### Mediating Analysis

**Figure 1.**
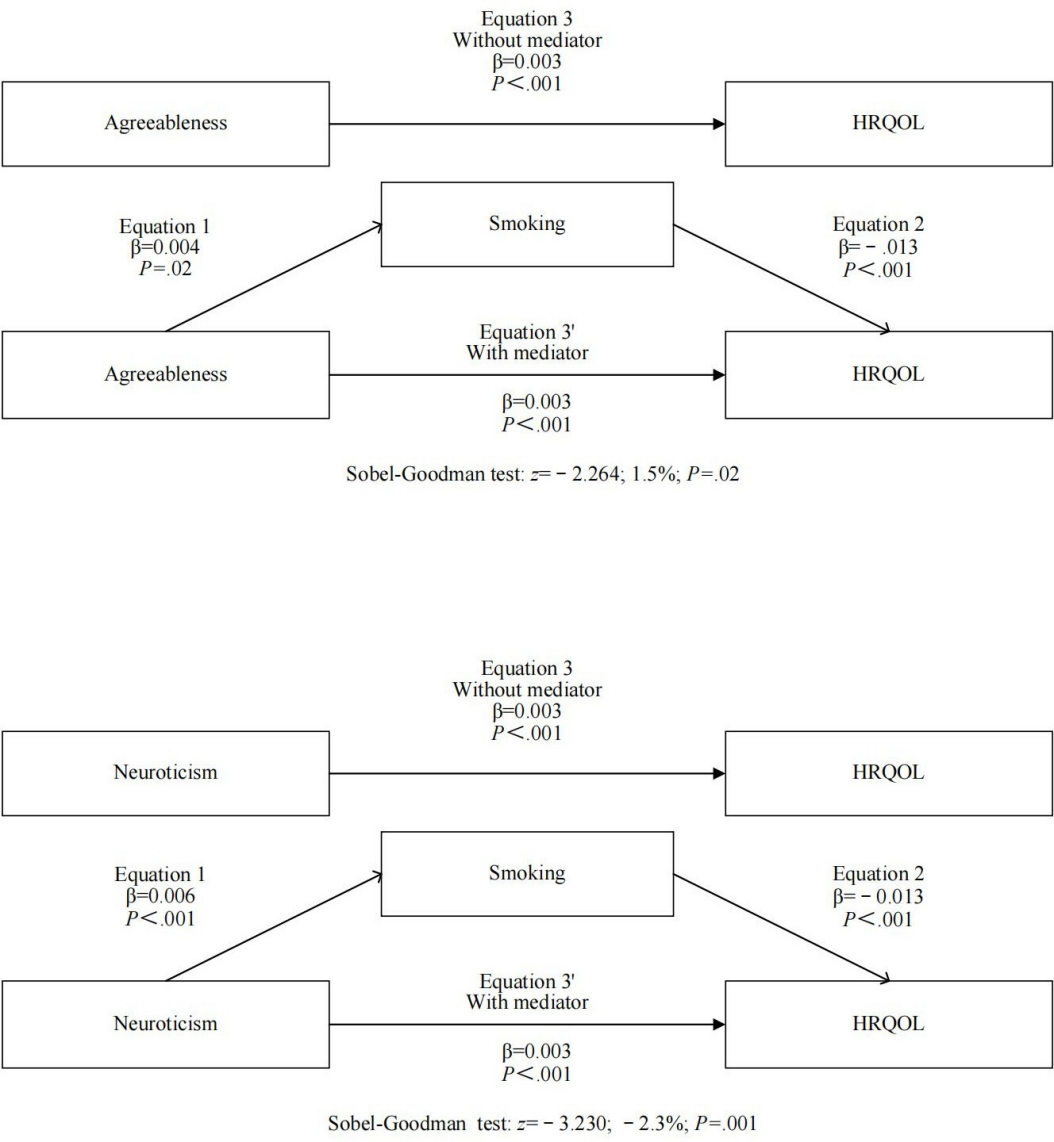
Mediating model of smoking in the association between Big Five personality traits and health-related quality of life (HRQOL).

Table 4 displayed the findings of the mediation analysis, indicating that smoking acted as a mediator for the impact of extraversion, agreeableness, and neuroticism on HRQOL. In terms of extraversion, there was a positive correlation between extraversion and smoking (β=.005; *P*=.002), whereas smoking showed a negative correlation with HRQOL (β=−.012; *P*<.001). Smoking mediated −13.3% of the effect HRQOL had on extraversion (*z*=−2.842; *P*=.004). In terms of agreeableness, there was a positive correlation between agreeableness and smoking (β=.004; *P*=.02), whereas smoking showed a negative correlation with HRQOL (β=−.013; *P*<.001). Additionally, agreeableness was positively correlated with HRQOL (β=.003; *P*<.001). Smoking mediated −1.5% of the effect HRQOL had on agreeableness (*z*=−2.264; *P*=.02). Neuroticism exhibited a positive correlation with smoking (β=.006; *P*<.001), whereas smoking showed a negative correlation with HRQOL (β=−.013; *P*<.001). Additionally, neuroticism displayed a positive correlation with HRQOL (β=.003; *P*<.001). Smoking mediated −2.3% of the effect HRQOL had on neuroticism (*z*=−3.230; *P*=.001). Figure 1 displays the ultimate mediation model.

**Table 4.** The mediating effect of smoking on Big Five personality traits and health-related quality of life (HRQOL), as explored by the Sobel-Goodman mediation test.

	Extraversion^a^		Agreeableness^a^		Conscientiousness^a^		Neuroticism^a^		Openness^a^	
	Value	*P* value	Value	*P* value	Value	*P* value	Value	*P* value	Value	*P* value
Big Five personality trait→smoking, β	0.005	.002	0.004	.02	0.002	.12	0.006	<.001	−0.000	.86
Smoking→HRQOL, β	−0.012	<.001	−0.013	<.001	−0.012	<.001	−0.013	<.001	−0.012	<.001
Indirect effect, β	−0.000	.004	−0.000	.02	−0.000	.13	−0.000	.001	0.000	.86
Direct effect, β	0.000	.24	0.003	<.001	0.002	<.001	0.003	<.001	−0.002	<.001
Total effect, β	0.000	.30	0.003	<.001	0.002	<.001	0.003	<.001	−0.002	<.001
Proportion of the total effect that is mediated	−0.133	—^b^	−0.015	—	−0.015	—	−0.023	—	−0.002	—
Sobel-Goodman mediation test	−2.842	.004	−2.264	.02	−1.531	.13	−3.230	.001	0.176	.86

a Adjusting for sex, age group, marital status, ethnicity, religion, political status, chronic disease, area of residence, family income, alcohol intake, education level, occupation, social status, family type, health literacy, self-efficacy, perceived stress, and perceived social support.

b Not applicable.

### Subgroup Analysis

**Figure 2.**
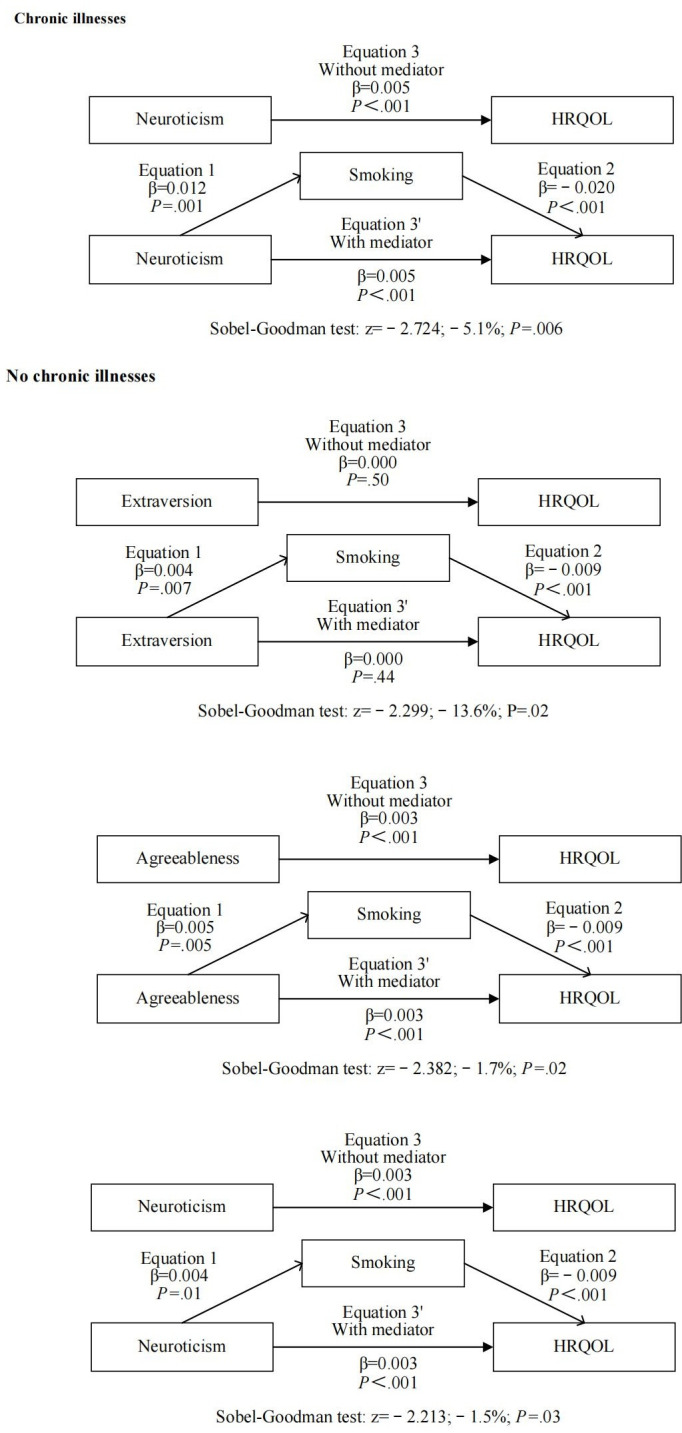
Subgroup analysis for mediating models of smoking in the association between Big Five personality traits and health-related quality of life (HRQOL).

In Table 5, the subgroup analysis revealed that smoking acted as a mediator between Big Five personality traits and HRQOL in individuals with chronic diseases. In particular, among individuals with chronic diseases, there was a positive correlation between neuroticism and smoking (β=.012; *P*=.001), whereas smoking showed a negative correlation with HRQOL (β=−.020; *P*<.001). Additionally, neuroticism exhibited a positive correlation with HRQOL (β=.005; *P*<.001). Smoking mediated −5.1% of the effect HRQOL had on neuroticism (*z*=−2.724, *P*=.006). Smoking mediated –13.6% of the effect HRQOL had on extraversion (*z*=−2.299; *P*=.02), –1.7% of the effect HRQOL had on agreeableness (*z*=−2.382; *P*=.02), and –1.5% of the effect HRQOL had on neuroticism (*z*=−2.213; *P*=.03) among the population without chronic illnesses. The extent to which smoking behavior mediated the relationship between agreeableness and HRQOL was slightly greater than that of neuroticism, whereas smoking behavior was the least relevant on the relationship between extraversion and HRQOL. Figure 2 displays the ultimate mediation model for the subgroup of the population with chronic diseases.

**Table 5.** Subgroup analysis of mediation models for Big Five personality traits associated with health-related quality of life (HRQOL), as mediated by smoking.

Subgroup		Extraversion^a^		Agreeableness^a^		Conscientiousness^a^		Neuroticism^a^		Openness^a^	
		Value	*P* value	Value	*P* value	Value	*P* value	Value	*P* value	Value	*P* value
**With chronic disease**											
	Big Five personality traits→smoking, β	0.007	.03	0.001	.69	−0.003	.44	0.012	.001	0.005	.16
	Smoking→HRQOL, β	−0.019	<.001	−0.019	<.001	−0.019	<.001	−0.020	<.001	−0.019	<.001
	Indirect effect, β	−0.000	.051	−0.000	.69	0.000	.45	−0.000	.006	−0.000	.18
	Direct effect, β	0.001	.26	0.004	<.001	0.003	.01	0.005	<.001	−0.001	.35
	Total effect, β	0.001	.32	0.004	<.001	0.003	.01	0.005	<.001	−0.001	.31
	Proportion of the total effect that is mediated	−0.133	—^b^	−0.006	—	0.018	—	−0.051	—	0.083	—
	Sobel-Goodman test	−1.948	.051	−0.397	.69	0.760	.45	−2.724	.006	−1.328	.18
**Without chronic disease**											
	Big Five personality traits→smoking, β	0.004	.007	0.005	.005	0.003	.04	0.004	.01	−0.002	.25
	Smoking→HRQOL, β	−0.009	<.001	−0.009	<.001	−0.009	<.001	−0.009	<.001	−0.009	<.001
	Indirect effect, β	−0.000	.02	−0.000	.02	−0.000	.07	−0.000	.03	0.000	.26
	Direct effect, β	0.000	.44	0.003	<.001	0.001	.002	0.003	<.001	−0.002	<.001
	Total effect, β	0.000	.50	0.003	<.001	0.001	.003	0.003	<.001	−0.002	<.001
	Proportion of the total effect that is mediated	−0.136	—	−0.017	—	−0.023	—	−0.015	—	−0.008	—
	Sobel-Goodman test	−2.299	.02	−2.382	.02	−1.841	.07	−2.213	.03	1.121	.26

a Adjusting for sex, age group, marital status, ethnicity, religion, political status, registered permanent residence, family income, alcohol intake, education level, work status, social status, household type, health literacy, self-efficacy, perceived stress, and perceived social support.

b Not applicable.

## Discussion

### Principal Findings

Prior research has established robust connections between tobacco use and HRQOL [44], along with the correlations between personality traits and both smoking and HRQOL [45,46]. Nevertheless, there has been no research conducted so far that has investigated the correlation among these 3 variables. The impact of smoking on the relationship between the Big Five personality traits and HRQOL was investigated in our research. We used data from a nationwide cross-sectional survey conducted in China, which encompassed 32 provinces, including autonomous regions and centrally governed municipalities. Through the process of mediation decomposition, we were able to provide additional evidence that smoking mediates the relationship between Big Five personality traits and HRQOL. The results of our study indicate that there is not only a direct correlation between Big Five personality traits and HRQOL but also an indirect correlation through smoking. Subgroup analyses for people with chronic conditions were also carried out.

The study findings indicated that extraversion, agreeableness, and neuroticism have a positive correlation with HRQOL, whereas openness has a negative correlation. Previous research has demonstrated that the Big Five personality traits impact well-being, life satisfaction, and self-identity, and thus HRQOL, through different personality traits [46]. People who possess elevated levels of extraversion and agreeableness have a greater tendency to form enduring and beneficial social connections, which can help reduce psychological strain and feelings of isolation [22,23]. Moreover, they possess a sunnier perspective on existence and exhibit enhanced resilience in the face of life’s obstacles and stresses [47]. Surprisingly, our research discovered a positive correlation between high neuroticism and HRQOL, which contradicts previous study results. There is a widespread belief that elevated neuroticism is linked to feelings of anxiety, depression, and various other negative emotions [48], ultimately resulting in a diminished quality of life [24,49]. However, according to the theory of healthy neuroticism, people with healthy neurotic traits aim for flawlessness and exhibit a heightened state of vigilance and self-reflection toward detrimental actions. As a result, their chances of encountering physical and psychological health issues are reduced [25,26]. Conversely, individuals who possess elevated levels of openness are not constrained by conventional notions and routines, and they might experience remorse for impulsive or erroneous behaviors [50]. Excessive attention to and maintenance of interpersonal relationships can also diminish life satisfaction and happiness [51].

Furthermore, our study provided evidence for the favorable correlations among extraversion, agreeableness, and neuroticism personality traits with smoking. Personality traits that promote social interactions, such as being friendly and reliable, are linked to high levels of extraversion and agreeableness [52]. As smoking is often perceived as a social behavior in some settings, these individuals may be more susceptible to social influence to start or quit smoking [53,54]. Conversely, people with elevated levels of neuroticism have a greater susceptibility to anxiety and stress [55]. Since nicotine can provide temporary relief from these emotions [56], individuals with high neuroticism may be inclined to use smoking as a coping mechanism, increasing their likelihood of initiating smoking or becoming regular smokers.

The act of smoking has been associated with a decrease in different aspects of HRQOL and the emergence of multiple long-term illnesses [57]. The results of our study indicated a consistent negative correlation between smoking and HRQOL, which was observed in both the entire population and the subgroup with chronic diseases, aligning with previous research. Furthermore, our research suggests that smoking mediates the relationship between extraversion, agreeableness, and neuroticism personality traits and HRQOL. High levels of extraversion and agreeableness are positively correlated with HRQOL, but they are also strongly associated with smoking, as these personality types are more likely to smoke due to a need for social interaction [58]. Smoking has a more significant correlation with HRQOL compared to the favorable correlations of high extraversion and agreeableness, thus smoking’s mediation weakens this positive effect. Furthermore, our research discovered that smoking can mediate the positive impact of highly healthy neuroticism on HRQOL. Prior research demonstrated that high neuroticism can be positively associated with HRQOL by promoting “healthy neuroticism” or introspection; it is also strongly associated with smoking, as people with high neuroticism are more likely to become dependent on tobacco for anxiety relief and experience symptoms of tobacco dependence [32]. The negative effect of smoking on HRQOL is greater than that of high neuroticism. Due to the mediation of smoking, the positive effect of high neuroticism on HRQOL is also weakened.

The findings from the subgroup analysis additionally indicate that smoking plays a mediate role in connecting neuroticism and HRQOL in the population with chronic diseases. However, the mediating effect in the population with chronic diseases remains similar to that of the overall population. The different mediating effects of personality traits in the populations with and without chronic diseases may be due to several factors. For example, the act of smoking is a major contributor to long-term health conditions, and people who have chronic illnesses may experience mental health issues such as neuroticism and anxiety due to their ailment. Furthermore, persistent illnesses frequently necessitate extended periods of therapy and medication, potentially leading to the formation of a neurotic character trait [59]. The findings of this research suggest that although the influence of mediation was minimal for certain traits, the Sobel-Goodman test produced noteworthy outcomes, indicating the existence of mediated routes. Considering the limited impact magnitudes, it is conceivable that alternative mechanisms are at play.

To summarize, our study supports the notion that smoking acts as a mediator in the relationship between the Big Five personality traits and HRQOL. Therefore, using a single tobacco control plan for the entire community may not be the best course of action; instead, tailored smoking cessation tactics based on various personality qualities can be taken into consideration. For individuals with high levels of extraversion and agreeableness, interventions such as smoking cessation environments; legislation; and support from partners, friends, or support groups can greatly increase the chances of successfully quitting smoking [60]. Providing emotional support, actively listening to their emotions and uncertainties, and assisting them in discovering suitable emotion management methods such as deep breathing and relaxation exercises to manage mood fluctuations during the process of quitting smoking could potentially yield greater advantages for individuals exhibiting elevated levels of agreeableness [61]. For people with high neuroticism, it is more important to promote self-reflection among people with high neuroticism and shape healthy neuroticism by sharing the dangers of smoking and health knowledge; provide anxiety management techniques such as deep breathing, meditation, or relaxation training to help them cope with anxiety and stress during the process of quitting smoking; and emphasize internal factors such as self-efficacy in interventions [62,63]. It is noteworthy that the correlation coefficients between personality traits and HRQOL in this study were small, and that HRQOL may provide a critical research perspective not from a clinical but from a psychosocial point of view, as HRQOL covers a wealth of information and personality traits are a potential factor influencing HRQOL. We initially explored the pathway through which Big Five personality traits influences individual HRQOL, and this pathway does exist. In addition, the mechanisms by which Big Five personality traits acts on HRQOL may be complex, and some mediating effects may be overshadowed by direct effects.

### Limitation

Although this study revealed a mediating role of smoking in the relationship between Big Five personality traits and HRQOL, it is important to acknowledge the existence of certain constraints that need to be considered. First, this study has the inherent limitations of cross-sectional studies in inferring causality. Because a cross-sectional study is conducted at a specific point in time, it can only reveal correlations between variables and cannot directly determine causality. Thus, although our cross-sectional study found associations between Big Five personality traits, smoking, and HRQOL, these results were not sufficient to suggest a causal relationship between them. Future research can explore the potential reciprocal association between the Big Five personality traits and HRQOL using longitudinal and prospective studies, thereby further validating and explaining our findings. Second, the correlation coefficients and mediating effects of our study were not very large, and further exploration needs to be made in the future as to exactly how Big Five personality traits affect HRQOL and how smoking mediates the relationship between Big Five personality traits and HRQOL. Finally, since all variables were reported by the participants themselves, there is a possibility of recall and cognitive biases being present, which could impact the precision of factors associated with health and personality. Furthermore, the formation of an individual’s character requires a significant amount of time, and as one matures, their character tends to become more steadfast and influenced by their surroundings. As a result, personality scores may have some bias in their immediate outcomes.

### Conclusion

This study demonstrated that smoking mediates the relationship between extraversion, agreeableness, and neuroticism personality traits and HRQOL. Additionally, smoking can mediate the effect neuroticism have on HRQOL in a population with chronic illnesses. In the future, when creating tobacco control strategies, it is important to consider the impact of personality, as suggested by these findings. We hope that our study will contribute to increasing the global smoking cessation rate and reducing the incidence of chronic diseases caused by smoking. This could assist in the advancement of campaigns promoting smoke-free initiatives and aid in the creation of a healthier and smoke-free atmosphere.

## Supplementary material

10.2196/51416 Multimedia Appendix 1 Health-related quality of life (HRQOL) questions and scores.

10.2196/51416 Multimedia Appendix 2 Variable description.

10.2196/51416 Multimedia Appendix 3 The covariate selection process (step 1): analyzing the relationship between each covariate and health-related quality of life (HRQOL).

10.2196/51416 Multimedia Appendix 4 The covariate selection process (step 2): covariates were introduced to the basic model and removed from the complete model to observe the change in the regression coefficients for smoking, extraversion, agreeableness, conscientiousness, neuroticism, and openness.

## Abbreviations

BFI: Big Five Inventory
HIS: health state index
HRQOL: health-related quality of life
