# Supplementary material for: The Association Between Personality Traits and Health-Related Quality of Life and the Mediating Role of Smoking: Nationwide Cross-Sectional Study
Source: JMIR Public Health Surveill. 2024 Jul 5;10:e51416. doi: 10.2196/51416 (PMC11240240; doi:10.2196/51416)
Supplement: Multimedia Appendix 1 [file publichealth-v10-e51416-s001.docx]

**Multimedia Appendix 1.** Health-related quality of life (HRQOL) Questions and Score.

| **Item number** | **Dimensions** | **Items** | **Mean±SD** |
| --- | --- | --- | --- |
| 1 | Action Capability | I walk around without difficulty.  I have a little trouble getting around.  I have moderate difficulty moving around.  I have serious difficulty moving around.  I can't move around. | 1.14 ± 0.52 |
| 2 | Self-Care | I have no difficulty bathing or dressing myself.  I have a little difficulty in bathing or dressing myself.  I have moderate difficulty bathing or dressing myself.  I have serious difficulty bathing or dressing myself.  I can't shower or dress myself. | 1.10 ± 0.47 |
| 3 | Daily Activities | I have no difficulty performing daily activities.  I have a little difficulty performing daily activities.  I have moderate difficulty performing daily activities.  I have serious difficulty performing daily activities.  I am unable to perform daily activities. | 1.12 ± 0.47 |
| 4 | Pain Or Discomfort | I have no pain or discomfort.  I have a little pain or discomfort.  I have moderate pain or discomfort.  I have severe pain or discomfort.  I have very severe pain or discomfort. | 1.28 ± 0.61 |
| 5 | Anxiety Or Depression | I am not anxious or depressed.  I have a little anxiety or depression.  I have moderate anxiety or depression.  I have severe anxiety or depression.  I have very severe anxiety or depression. | 1.35 ± 0.68 |
