# Supplementary material for: The Association Between Personality Traits and Health-Related Quality of Life and the Mediating Role of Smoking: Nationwide Cross-Sectional Study
Source: JMIR Public Health Surveill. 2024 Jul 5;10:e51416. doi: 10.2196/51416 (PMC11240240; doi:10.2196/51416)
Supplement: Multimedia Appendix 2 [file publichealth-v10-e51416-s002.docx]

# Table S2 is shown in use on **page 9** of the manuscript.

# Table S2. Variable description.

| Variable name | Definition or code |
| --- | --- |
| **Dependent variable** |  |
| Health-related quality of life (HRQOL) | The health index refers to the assessment of health from five dimensions: mobility, self-care ability, daily activity ability, pain/discomfort, and anxiety/depression through the classic scale of quality of life (EQ-5D-5L). Each dimension has five levels of responses (no problem, minor problem, moderate problem, severe problem, extreme problem/inability to score), scored using a 5-point Likert scale for scoring, assigning a summary index score to each health state by its social preference weighting. The health status index usually goes from less than 0 (where 0 is equivalent to the health status value of death, and a negative value indicates a worse state than death) to 1 (the value of complete health), according to which the index is converted to obtain the final health index value, the larger the index indicates the higher the health utility of the respondent. |
| **Mediation variable** |  |
| Smoke habit | It indicates the respondent’s current smoking habit. 0=Never smoked or used to smoke but have now quit; 1=Currently smoking. |
| **Independent variables** |  |
| Big five personality | It was measured using the Big Five Classic Inventory of Personality (BFI-10), which consists of 10 entries with 5 dimensions, using a 5-point Likert scale. The five dimensions were extraversion, agreeableness, conscientiousness, neuroticism, and openness. |
| **Covariates** |  |
| Age group | It indicates the age group of the respondents. 1=Ages twelve to seventeen; 2=Ages Eighteen to fifty-nine; 3= Greater than or equal to 60 years old. |
| Sex | 1=male; 2=female. |
| Marital status | 1=Never Married; 2=Married, including the first marriage with a spouse, and then remarried with a spouse; 3=Divorce; 4=Widowed. |
| Ethnicity | In the People's Republic of China, the 55 legal nationalities other than the Han, the main nationality, are all ethnic minorities.0= Han nationality ;1=Ethnic Minorities |
| Religion | 0=None; 1=Yes. |
| Political status | Political refers to political appearance, indicating the respondents' political affiliation, is the most direct reflection of a person's political identity, refers to the political parties, political groups in which a person participates. It is a simplified version of the codes for political affiliation issued by the National Bureau of Standards of the People's Republic of China. 1=Member of the Communist Party of China, including preparatory members of the Communist Party of China; 2=Member of the Communist Youth League; 3=Other party affiliation; 4=Mass. |
| Area of residence | Residence indicates the household living region and is defined by  National Bureau of Statistics of the People's Republic of China. 1=urban; 2=rural. |
| Education level | Education level is a simplified version of the 1997 International Standard Classification of Education (ISCED-97) code. 1 = Primary education and below; 2 = Lower to upper secondary education; 3 = Tertiary education (tertiary and above). |
| Occupation | Work status indicates the current occupational status of the respondent. 1=Employed, 2=Student, 3=Retired, 4=Unemployed or freelance, 5=Unemployed, non-working, or jobless. |
| Family type | Family type indicates the respondent's current family status. 1=Backbone family, which is a family consisting of only two spouses, husband and wife; 2=Core family, which is a family consisting of parents and unmarried children; 3=Conjugal family, which is a family consisting of parents and married children; 4=Other, including families consisting of parents and more than two married children or siblings married without joint families; single-parent families; DINK (double income no kids); intergenerational families; single families; reconstituted families; cohabiting families; homosexual families, etc. |
| Family income | Family income is the respondent's current monthly per capita family income, which is calculated as total household income divided by the total number of people, where 1=Low (less than $3,000), 2=Moderate (between $3,001 and $5,000), and 3=High (more than $5,001). |
| Alcohol intake | Alcohol intake indicates whether the respondent drank alcohol in the past or at present. 0=Never drank, 1=Always drank, 2=Drank in the past but not now, 3=Did not drink in the past but now drink. |
| Chronic | Chronic diseases diagnosed by doctors. 0=No chronic disease; 1=Suffer from one or more chronic disease. |
| Health literacy | It is a health literacy score measured by combining the HLS-SF12 scale for the 2021 international introduction and the 2022 entry condensation. The questionnaire contains a total of 9 items in 3 dimensions of health care, disease prevention and health promotion. The 5-point Likert scale method is used for scoring, the total score range is 9~36 points, the higher the score, the higher the health literacy. |
| Perceived Stress | It is measured using the classical scale of perceived pressure (PSS-4), which has a total of 4 items, using the 5-point Likert scale method for scoring, with a total score range of 0~16 points, the higher the score indicates that the respondent is more stressed. |
| Perceived social support | It is measured using the 2022 Entry Lite Comprehension Social Support Scale (PSSS), which is divided into 3 dimensions of family support, friend support and other support, with a total of 3 items, and is assigned using the 7-point Likert scale. The total score ranges from 0~21 points, with higher scores indicating the higher the level of total social support felt by respondents. |
| Self-efficacy | It is measured using the New General Self-Efficacy Energy Scale (NGSES) of the 2022 Articles Condensed Version, which is divided into 3 dimensions of level or degree, intensity and universality, a total of 3 items, using the 5-point Likert scale for scoring, the total score range is 0~12 points, the higher the score indicates the stronger the respondent's self-efficacy. |
| Social status | Social status is a self-rating of how respondents think their family is in social status. The score ranges from 1 to 7 points, with 1 representing the lowest social status and 7 representing the uppermost social status. |
