# Supplementary material for: The Association Between Personality Traits and Health-Related Quality of Life and the Mediating Role of Smoking: Nationwide Cross-Sectional Study
Source: JMIR Public Health Surveill. 2024 Jul 5;10:e51416. doi: 10.2196/51416 (PMC11240240; doi:10.2196/51416)
Supplement: Multimedia Appendix 3 [file publichealth-v10-e51416-s003.docx]

# Multimedia Appendix 3. The Selection Process of Covariates: Step 1 - Analyzing the Relationship Between the Covariate and Y (Y=HRQOL) One by One.

| Covariates | term | beta | Se. | 95%CI Low | 95%CI Upp | P.value |
| --- | --- | --- | --- | --- | --- | --- |
| Marital status | Married | 0.0130 | 0.0016 | 0.0099 | 0.0161 | < .0001 |
|  | Divorce | -0.0308 | 0.0050 | -0.0407 | -0.0209 | < .0001 |
|  | Widowed | -0.0425 | 0.0045 | -0.0514 | -0.0336 | < .0001 |
| Nation | Minorities | -0.0103 | 0.0023 | -0.0149 | -0.0057 | < .0001 |
| Religion | Yes | -0.0222 | 0.0035 | -0.0290 | -0.0155 | < .0001 |
| Political | member of the Communist Youth League | -0.0022 | 0.0023 | -0.0067 | 0.0024 | .3468 |
|  | Other parties | -0.0385 | 0.0081 | -0.0545 | -0.0225 | < .0001 |
|  | The masses | 0.0058 | 0.0020 | 0.0020 | 0.0097 | .0030 |
| Registered permanent residence | Rural | -0.0035 | 0.0014 | -0.0061 | -0.0008 | .0103 |
| Family income | Moderate | 0.0119 | 0.0017 | 0.0086 | 0.0151 | < .0001 |
|  | High | 0.0079 | 0.0016 | 0.0048 | 0.0111 | < .0001 |
| Alcohol intake | All the time | -0.0199 | 0.0020 | -0.0238 | -0.0160 | < .0001 |
|  | Used to drink now not drink | -0.0319 | 0.0023 | -0.0364 | -0.0273 | < .0001 |
|  | The past does not drink now drink | -0.0392 | 0.0029 | -0.0449 | -0.0336 | < .0001 |
| Chronic disease | Yes | -0.0428 | 0.0016 | -0.0460 | -0.0396 | < .0001 |
| Education | Middle school and junior college | 0.0084 | 0.0021 | 0.0042 | 0.0126 | .0001 |
|  | College degree or above | 0.0008 | 0.0022 | -0.0036 | 0.0052 | .7054 |
| Work | Student | -0.0146 | 0.0018 | -0.0182 | -0.0110 | < .0001 |
|  | Retirement | -0.0224 | 0.0030 | -0.0283 | -0.0165 | < .0001 |
|  | No regular occupation | -0.0035 | 0.0023 | -0.0080 | 0.0009 | .1229 |
|  | Unemployed | -0.0168 | 0.0027 | -0.0221 | -0.0116 | < .0001 |
| Social | - | 0.0048 | 0.0005 | 0.0038 | 0.0058 | < .0001 |
| Household | Core family | 0.0080 | 0.0019 | 0.0042 | 0.0117 | < .0001 |
|  | Main family | 0.0052 | 0.0023 | 0.0007 | 0.0097 | .0236 |
|  | Other forms of family | 0.0314 | 0.0025 | -0.0362 | -0.0265 | < .0001 |
| Perceived Stress | - | -0.0074 | 0.0003 | -0.0079 | -0.0069 | < .0001 |
| Perceived social support | - | 0.0045 | 0.0002 | 0.0041 | 0.0048 | < .0001 |
| Self-efficacy | - | 0.0067 | 0.0003 | 0.0062 | 0.0073 | < .0001 |
| Health literacy | - | 0.0034 | 0.0001 | 0.0031 | 0.0036 | < .0001 |
