# Supplementary material for: The Association Between Personality Traits and Health-Related Quality of Life and the Mediating Role of Smoking: Nationwide Cross-Sectional Study
Source: JMIR Public Health Surveill. 2024 Jul 5;10:e51416. doi: 10.2196/51416 (PMC11240240; doi:10.2196/51416)
Supplement: Multimedia Appendix 4 [file publichealth-v10-e51416-s004.docx]

**Multimedia Appendix 4.** The Selection Process of Covariates. Step 2 - Covariates Were Introduced into The Basic Model and Removed from The Complete Model to Observe the Change of the Regression Coefficient of X (X=Smoke, Extraversion, Agreeableness, Conscientiousness, Neuroticism, Openness).

| Covariates | Basic model | | | | | | Complete model | | | | | | Selected |
| --- | --- | --- | --- | --- | --- | --- | --- | --- | --- | --- | --- | --- | --- |
|  | Smoke | Extraversion | Agreeableness | Conscientiousness | Neuroticism | Openness | Smoke | Extraversion | Agreeableness | Conscientiousness | Neuroticism | Openness |  |
| Marital | -0.0294 | 0.0040 | 0.0074 | 0.0058 | 0.0082 | 0.0009 | -0.0149 | 0.0007 | 0.0027 | 0.0007 | 0.0032 | -0.0013 | Yes |
| Nation | -0.0280 | 0.0040 | 0.0078 | 0.0062 | 0.0085 | 0.0002 | -0.0150 | 0.0007 | 0.0027 | 0.0008 | 0.0032 | -0.0013 | Yes |
| Religion | -0.0279 | 0.0040 | 0.0078 | 0.0062 | 0.0085 | 0.0002 | -0.0150 | 0.0007 | 0.0027 | 0.0008 | 0.0033 | -0.0013 | Yes |
| Political | -0.0291 | 0.0041 | 0.0078 | 0.0062 | 0.0084 | 0.0006 | -0.0150 | 0.0007 | 0.0027 | 0.0007 | 0.0032 | -0.0013 | Yes |
| Hukou | -0.0280 | 0.0040 | 0.0078 | 0.0062 | 0.0085 | 0.0001 | -0.0150 | 0.0007 | 0.0027 | 0.0008 | 0.0032 | -0.0013 | Yes |
| Income | -0.0276 | 0.0039 | 0.0078 | 0.0061 | 0.0084 | 0.0000 | -0.0151 | 0.0008 | 0.0027 | 0.0008 | 0.0032 | -0.0013 | Yes |
| Drink | -0.0226 | 0.0044 | 0.0076 | 0.0060 | 0.0084 | 0.0006 | -0.0193 | 0.0004 | 0.0028 | 0.0009 | 0.0032 | -0.0015 | Yes |
| Chronic disease | -0.0241 | 0.0038 | 0.0078 | 0.0066 | 0.0081 | -0.0002 | -0.0164 | 0.0008 | 0.0026 | 0.0006 | 0.0035 | -0.0013 | Yes |
| Education | -0.0284 | 0.0040 | 0.0078 | 0.0062 | 0.0085 | 0.0003 | -0.0147 | 0.0008 | 0.0027 | 0.0008 | 0.0033 | -0.0014 | Yes |
| Work status | -0.0306 | 0.0041 | 0.0076 | 0.0059 | 0.0084 | 0.0008 | -0.0143 | 0.0007 | 0.0027 | 0.0009 | 0.0032 | -0.0014 | Yes |
| Social status | -0.0278 | 0.0036 | 0.0076 | 0.0058 | 0.0082 | 0.0001 | -0.0150 | 0.0008 | 0.0027 | 0.0008 | 0.0032 | -0.0013 | Yes |
| Household type | -0.0256 | 0.0039 | 0.0073 | 0.0058 | 0.0083 | 0.0004 | -0.0160 | 0.0007 | 0.0028 | 0.0008 | 0.0032 | -0.0014 | Yes |
| Perceived Stress | -0.0264 | 0.0014 | 0.0046 | 0.0025 | 0.0049 | -0.0009 | -0.0152 | 0.0019 | 0.0041 | 0.0025 | 0.0050 | -0.0008 | Yes |
| Perceived social support | -0.0240 | 0.0021 | 0.0050 | 0.0040 | 0.0065 | -0.0009 | -0.0152 | 0.0008 | 0.0030 | 0.0008 | 0.0033 | -0.0013 | Yes |
| Self-efficacy | -0.0245 | 0.0020 | 0.0055 | 0.0035 | 0.0062 | -0.0011 | -0.0151 | 0.0008 | 0.0027 | 0.0008 | 0.0033 | -0.0012 | Yes |
| Health literacy | -0.0232 | 0.0027 | 0.0060 | 0.0051 | 0.0071 | -0.0017 | -0.0165 | 0.0009 | 0.0030 | 0.0008 | 0.0034 | -0.0008 | Yes |
